# The Population Growth Consequences of Variation in Individual Heterozygosity

**DOI:** 10.1371/journal.pone.0019667

**Published:** 2011-05-18

**Authors:** Martina M. I. Di Fonzo, Fanie Pelletier, T.H. Clutton-Brock, Josephine M. Pemberton, Tim Coulson

**Affiliations:** 1 Department of Life Sciences, Imperial College London, Ascot, Berkshire, United Kingdom; 2 Institute of Zoology, Zoological Society of London, London, United Kingdom; 3 Department of Zoology, University of Cambridge, Cambridge, United Kingdom; 4 Institute of Evolutionary Biology, University of Edinburgh, Edinburgh, United Kingdom; University of Zürich, Switzerland

## Abstract

Heterozygosity has been associated with components of fitness in numerous studies across a wide range of taxa. Because heterozygosity is associated with individual performance it is also expected to be associated with population dynamics. However, investigations into the association between heterozygosity and population dynamics have been rare because of difficulties in linking evolutionary and ecological processes. The choice of heterozygosity measure is a further issue confounding such studies as it can be biased by individual differences in the frequencies of the alleles studied, the number of alleles at each locus as well as the total number of loci typed. In this study, we first examine the differences between the principal metrics used to calculate heterozygosity using long-term data from a marked population of Soay sheep (*Ovis aries*). Next, by means of statistical transformation of the homozygosity weighted by loci index, we determine how heterozygosity contributes to population growth in Soay sheep by modelling individual contributions to population growth (*p_t(i)_*) as a function of several covariates, including sex, weight and faecal egg count – a surrogate of parasitic nematode burden in the gut. We demonstrate that although heterozygosity is associated with some components of fitness, most notably adult male reproductive success, in general it is only weakly associated with population growth.

## Introduction

A positive association between neutral marker heterozygosity and individual performance (also known as a heterozygosity-fitness correlation; HFC) has been reported by numerous studies. In particular, heterozygosity has been found to affect components of individual fitness including survival [1], [2], breeding success [3], [4], [5], disease resistance [6], parasite resistance [7], territory size [8], birdsong complexity [8], growth rate [9], developmental stability [10] and quantitative traits such as birth weight [1], [2]. Since individual heterozygosity influences individual performance, it is also expected to affect population dynamics e.g. [11], [12].

Identifying the population dynamic signature of fluctuations in heterozygosity is challenging because until recently it has not been possible to easily link individual and population-level processes due to the traditional view that they operate over different time scales. This issue has been resolved by Pelletier *et al.* [13], who put forward a new statistical technique which links trait variation and population growth. Prior to this development, heterozygosity has still been associated with extinction risk [11] and population growth [12] in a meta-population of Glanville fritillary butterflies, as well as in the population recoveries of inbred bighorn sheep [14], wolves [15], [16] and adders [17], through a range of analyses at the population-level.

A second challenge when investigating the individual or population level consequences of heterozygosity is how to transform heterozygosity to generate a statistically comparable estimator of multi-locus heterozygosity. Specifically there are three sources of bias that may arise within heterozygosity measures , which require correction: differences in (a) the number of alleles at each locus, (b) the frequency of different alleles at each locus and (c) the number of loci at which an individual is typed if not all individuals have been typed at all loci [18]. The simplest heterozygosity measure is individual multi-locus heterozygosity (*MLH*), defined as the proportion of heterozygous loci within an individual [19]. The advantage of using this measure is that it is extremely straightforward, however it does not correct for differences in number and frequency of alleles (i.e. expected heterozygosity) between loci [18]. A method for calculating heterozygosity, which takes into account the differences in mean heterozygosity between individuals as a function of the panel of typed loci, is standardised individual heterozygosity (*H_s_*; [7]). Another technique which corrects for heterozygosity at each locus, given allele frequencies, is the homozygosity weighted by loci (*HL*) index [18]. It is an advance on *H_s_* as it weighs the contribution of each locus to the homozygosity value depending on its expected heterozygosity [18]. Internal relatedness (IR; [3]) is a further measure of heterozygosity, which not considered in this study due to asymmetries in its treatment of allele frequency (critiqued in [18]).

Pelletier *et al.*'s [13] method can be employed to assess the link between individual differences in heterozygosity and population growth by determining the proportion of variation in individual contributions to population growth (*p_t(i)_*; [20]) explained by heterozygosity in a multivariate, statistical framework. An individual's contribution to growth over a time step is estimated by calculating the difference between observed population growth and population growth calculated with the contribution of the focal individual via survival (*S_t(i)_*) and recruitment (*F_t(i)_*) removed [20]. Specifically,
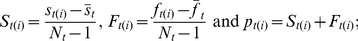
(1)where *s_t(i)_* and *f_t(i)_* are survival and recruitment of individual *i* at time *t* and 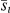 and 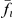 define mean survival and recruitment across all *N* individuals in the population. Pelletier *et al.* [13] decomposed *p_t(i)_* to examine how body mass variation influenced population growth within this population using univariate analyses; the method has not been applied before to other populations, extended to the multivariate case or indeed to heterozygosity.

In this paper we address three questions. First, we examine how *MLH*, *H_s_* and *HL* differ. Second, having identified increases in the variance of each of these measures with increasing marker number, we apply a normalising transformation to make our heterozygosity data statistically comparable. Third, we examine how individual heterozygosity, estimated using the normalised homozygosity weighted by loci (*HL*) index, contributes to population growth in the unmanaged population of Soay sheep (*Ovis aries* L.) in St Kilda, Scotland. We do this by extending Pelletier *et al.*'s [13] technique of modelling individual contributions to population growth as a function of multiple covariates. We report that variation in normalised *HL* is weakly associated with population growth in most demographic classes, apart from that of adult males.

## Methods

### Study system

The Soay sheep is a primitive domestic breed that is thought to have existed unmanaged on the St Kilda archipelago, Scotland, for the past two to three thousand years [21]. The present population, on the island of Hirta (638 ha), is the result of the introduction of 107 individuals from the neighbouring island of Soay (99 ha) in 1932 [22].

Since 1985, Soay sheep within the Village Bay area of Hirta (ca. 175 ha) have been closely monitored [23], [24]. Individuals are tagged soon after birth, regularly recaptured and followed throughout life. Their birth and death dates and breeding success are recorded, along with information regarding morphometric traits (including body weight and faecal egg count, referred to as FEC) and they are genotyped for several microsatellite markers, primarily for paternity analysis. Here, we define the sheep year as running from August 1^st^ to July 31^st^, and recruitment is defined as the number of lambs an individual produced in April that are still alive in August of the same year. An unusual characteristic of this population is its unstable population dynamics [25], [26], with total population size fluctuating between approximately 600 and 2000 individuals. The Village Bay population represents approximately one third of the total island population [27], and experiences population fluctuations that are strongly correlated with those affecting the entire island [21], [22], [24]. Further details regarding the study site, methods used for data collection and previous research on this population can be found in Clutton-Brock and Pemberton [21].

The probability of survival following a population crash varies with age and sex [28], [29]. Mortality rates are higher in males than females and mature individuals have greater chances of survival than yearlings or lambs. Mortality rates also differ among mature individuals, being highest in prime-aged adults (2 to 6 years) and lower in senescent individuals (>6 years) [23], [28]. Separate analyses were conducted for males and females in each demographic class due to differences in survival between the sexes [30]. Prime-aged and senescent males were combined due to the small sample size of males over 6 years of age (n = 25). Hereafter this category will be known as adult males.

### Individual-level covariates

Lambs were assigned to mothers by field observations of maternal behaviours [21]. Fathers were assigned both by genotyping and using the likelihood-based inference program CERVUS 3.0 [31], with a confidence of 80% and a maximum of one mismatch between parents and offspring. To calculate heterozygosity we used the same genetic dataset. Between 1985 and 2008 a total of 4,543 individuals were screened at a panel of up to 42 loci, using the method detailed in Overall *et al.* [32]. After the omission of functional loci, 25 putatively neutral unlinked microsatellite loci were available for analysis, (shown in Table 1). Body weight (kg) measurements have been recorded every August since 1985, during the annual catch of resident sheep. Parasite load in a year was estimated by taking the mean of repeated individual strongyle faecal egg counts, determined using a modified McMaster technique [33]. Individual contributions to population growth (*p_t(i)_*), survival (*S_t(i)_*) and recruitment (*F_t(i)_*) were calculated from life history data, using the previously stated formula [20].

**Table 1:** Population data for all putatively neutral, unlinked microsatellite loci screened.

					Heterozygosity	Heterozygosity	HWE test (p-value)
Locus	Chromosome number	Number of Alleles	Cohorts screened (year groups)	Number of individuals Scored	Expected	Observed	
**AE54**	25	6	83-08	3147	0.629	0.613	0.199
**BL4**	3	5	80-99	1135	0.601	0.589	0.816
**BM1314**	22	8	83-99	1271	0.802	0.784	0.402
**BM203**	26	11	84-99	1290	0.781	0.741	<0.001
**CP26**	4	5	79-08	4042	0.703	0.692	0.257
**FCB20**	2	7	83-08	3274	0.662	0.658	0.821
**FCB304**	19	4	85-07	4144	0.622	0.615	0.1348
**FCB48**	17	4	83-02	1741	0.497	0.501	0.326
**HH47**	18	6	83-08	3273	0.673	0.664	0.498
**INRA5**	10	8	83-08	3253	0.702	0.708	0.291
**JMP29**	24	4	83-08	3270	0.665	0.670	0.966
**JMP58**	26	5	83-08	3288	0.587	0.578	<0.001
**MAF209**	17	8	83-08	3149	0.728	0.724	0.917
**MAF33**	9	4	88-01	1114	0.376	0.343	<0.001
**MAF35**	23	4	85-94	4113	0.566	0.579	0.309
**MAF45**	X(PAR)	7	77-08	4145	0.735	0.732	0.158
**MAF65**	15	4	77-94	1252	0.488	0.518	<0.001
**MAF70**	4	6	83-08	3099	0.786	0.756	0.001
**MCM140**	6	6	83-08	3252	0.625	0.616	0.606
**MCM527**	5	7	83-08	3284	0.761	0.750	0.229
**RM106**	16	4	79-94	1217	0.456	0.454	0.959
**TGLA13**	2	6	83-08	3231	0.740	0.721	<0.001
**TGLA263**	1	7	83-08	3280	0.780	0.784	0.062
**TGLA53**	12	8	83-08	3250	0.659	0.652	0.038
**VH34**	3	5	79-08	4098	0.560	0.538	<0.001

FCB304 was excluded from analyses as its heterozygosity values were significantly correlated with two other loci. Individuals from cohorts prior to 1985 were retro-genotyped as candidate parents. HWE stands for Hardy-Weinberg Equilibrium.

### Calculating heterozygosity

Loci were first checked for deviations from Hardy-Weinberg equilibrium in CERVUS 3.0 [31] (Table 1). Next, homozygosity/heterozygosity was coded as a binary variable (0/1) for each locus at which an individual was typed. In order to check that heterozygosity was statistically independent between loci, Spearman rank correlations were performed between heterozygosity measures at each locus. To estimate individual heterozygosity across the selected panel of markers, we employed three distinct measures: multilocus heterozygosity (*MLH*; [19]), standardised heterozygosity (*H_s_*; [7]) and the homozygosity weighted by loci (*HL*) index [18].

Individual *MLH* is calculated by measuring the proportion of heterozygous loci within an individual. *H_s_* is estimated by dividing the proportion of heterozygous loci within an individual by the average of the population–level mean heterozygosities of each locus genotyped in that individual [7]. The *HL* measure is the residual between observed heterozygosity and expected heterozygosity given allele frequencies at each locus [18]. That is:
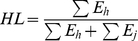
(2.1)where *E_h_* and *E_j_* are respectively the expected heterozygosities of the homozygous and heterozygous loci of an individual. Expected heterozygosity (*E*) is estimated by:
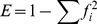
(2.2)where *f_i_* = the frequency of the i^th^ allele in the population [18].

Since the variance in all heterozygosity measures tended to decrease with the number of loci typed (see results), we calculated normalised estimates to make our loci statistically comparable. This ensured that mean heterozygosity across all individuals was zero and the standard deviation was 1, regardless of how many loci they were typed at. Normalisation was carried out using the following equation:
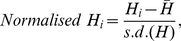
(3)where *H_i_* represents individual *i*'s heterozygosity measure, 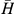 and *s.d.(H)* are respectively the mean and standard deviation of these measures across all individuals typed at the same number of loci as the focal individual.

### Contribution analyses

All analyses were carried out using R version 2.9.0 [34]. Multiple regressions of individual contributions to population growth (*p_t(i)_*, *S_t(i)_* and *F_t(i)_*) as a function of body weight, FEC, normalised *HL* and year were carried out for each demographic class. A reduced dataset was used for these analyses that only included individuals which had complete information for these traits (n = 4374). Year was fitted as a factor, and interactions between year and the individual covariates were also investigated. We used a backward model simplification procedure by first fitting saturated models. The models were then simplified by deleting non-significant terms. The r^2^ values of the minimum adequate models were used to describe the amount of variation in individual contributions to population growth explained by individual traits in each demographic class [13]. In order to define the amount of variation explained by normalised *HL* alone, the regressions were repeated with and without this term. The difference between the r^2^ values of the two models represented the proportion accounted for by individual differences in heterozygosity. An identical approach was used to obtain an estimate of the contribution of other model terms to population growth.

We established the amount of variation explained by individual traits in female and male contributions to population growth by multiplying the r^2^ of each age class by the proportion of the total female or male population within that age class. To describe the amount of variation across the whole population in individual contributions to population growth accounted for by individual traits we summed the products of the r^2^ values for each demographic class and the proportion of the population represented by that class.

We used multiple regressions throughout the analysis. The only age-classes for which the use of these models could be statistically problematic are adult males and prime-aged females because of repeated measures on individuals. Individual adult males were measured on average 1.71 times when both prime-aged and senescent individuals are included in the calculation. For prime-aged females, the average number of repeated observations was 2.27 times. A linear mixed effect model was fitted with ID as a random effect for prime-aged females to determine whether pseudoreplication was an issue in this analysis. The t-values of the minimum adequate model terms remained significant and estimates did not significantly change, indicating that pseudoreplication does not affect our results.

## Results

The majority of the loci which we used in our analysis are statistically heterozygosity-independent, with a few showing weak correlations between their homozygosity/heterozygosity values (specified respectively as 1/0). The heterozygosity at microsatellite marker loci FCB304 showed high statistical correlation with heterozygosity values of RM106 and a slight but significant correlation with MAF45 (Spearman's rank correlation; rho = 0.08, p<0.001 and rho = 0.099, p<0.05 respectively). Hence, FCB304 was excluded from the analyses in order to maintain a consistent assumption of loci independence in subsequent analyses.

**Figure 1:**
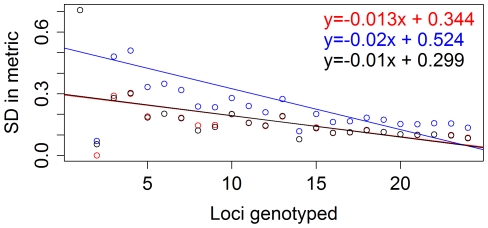
The bias in heterozygosity measures prior to normalisation. This is illustrated by the manner in which the standard deviation (SD) of the measures decreases with increasing number of genotyped markers (red = *MHL*, r^2^ = 0.476, F_1, 21_ = 19.05, p<0.001, n = 24; blue = *H_s_*, r^2^ = 0.33, F_1, 22_ = 10.84, p = 0.003, n = 24; and black = *HL*, r^2^ = 0.326, F_1, 22_ = 10.63, p = 0.004, n = 24). The trend lines indicate the linear regression between the SD in heterozygosity measures with increasing loci genotyped.

Individual multi-locus heterozygosity (*MLH*), standardised heterozygosity (*H_s_*) and homozygosity weighted by loci (*HL*) all showed a substantial decrease in variance as the number of markers at which an individual was genotyped increased (Figure 1). Such heteroscedasticity can easily be removed by normalising the measures to ensure that the mean heterozygosity measure across individuals typed at the same number of loci is zero and the standard deviation across them is one. Following normalisation, all measures of heterozygosity were qualitatively similar and correlated with each other (see Table S1). We decided to concentrate on normalised *HL* in subsequent analyses as it is an improvement over previous metrics since it takes into account differences in allele frequency. Normalised *HL* is a measure of homozygosity, thus lower values correspond to higher heterozygosity.

To evaluate the possibility that different demographic classes may be influenced by variation in heterozygosity to different extents, we next examined how normalised *HL* influenced *p_t(i)_*, *S_t(i)_* and *F_t(i)_* in male and female Soay sheep of different ages. We found normalised *HL* only contributing significantly to population growth through female prime-aged survival (*S_t(i)_*) and male lamb and adult overall contributions (*p_t(i)_*) and fecundities (*F_t(i)_*) (Table 2). Within the male section of the population, variation in normalised *HL* in an interaction with year explains approximately three times more variation in contributions to population growth via adult fecundity and 1.5 times more variation in contributions via lamb fecundity compared to that explained in prime-aged female survival (Tables 3–4). When normalised *HL* is considered on its own, it explains twice the amount of variation in individual contributions to population growth in adult male *p_t(i)_* and approximately the same amount of variation in lamb and adult fecundity compared to prime-aged female survival (being 0.13%). Within males, normalised *HL* in an interaction with year explains approximately twice as much variation in adult *p_t(i)_* and *F_t(i)_* compared with lamb *F_t(i)_*. Overall, the male age-sex class analyses show that normalised *HL* explains the least amount of variation compared to other traits. Within prime-aged females, normalised *HL* explains the least amount of variation in contributions to population growth out of all the traits, except for when it is considered in an interaction with year, where it explains slightly more (approx. 1%) than FEC in such an interaction.

**Table 2:** Minimum adequate models for the associations between *p_t(i)_*, *S_t(i)_* and *F_t(i)_* and their individual covariates.

Sex	Age-class	*pt(i)*	*St(i)*	*Ft(i)*
Female	Lambs	Weight*year+FEC*year	weight*year+FEC*year	weight*year
Female	Yearlings	Weight*year	Weight*year+FEC	Weight*year
Female	Prime-aged	Weight*year+FEC*year	Weight*year+FEC*year+heterozygosity*year	FEC*year
Female	Senescent	Weight*year+FEC*year	Weight*year+FEC*year	Weight*year
Male	Lambs	Weight*year+FEC*year+heterozygosity*year	Weight*year+FEC*year	Heterozygosity*year
Male	Yearlings	Weight*year	Weight+year	Weight*year
Male	Adults	Weight*year+FEC*year+heterozygosity	Year	Weight*year+FEC*year+heterozygosity

Significant interactions that include heterozygosity (normalised *HL*) are highlighted in bold. The asterisk represents the interactive and additive effects between covariates.

**Table 3:** Percentage of variation explained by individual covariates in individual contributions to growth within male age classes.

	Lambs	Lambs	Lambs	Yearlings	Yearlings	Yearlings	Adults	Adults	Adults
Models	*p_t(i)_*	*S_t(i)_*	*F_t(i)_*	*p_t(i)_*	*S_t(i)_*	*F_t(i)_*	*p_t(i)_*	*S_t(i)_*	*F_t(i)_*
**MAM**	**21.72**	**18.74**	**30.81**	**25.8**	**12.91**	**36.73**	**51.57**	**10.96**	**55.61**
- Year	**7.066**	**5.538**	**25.198**	**16.539**	**9.189**	**26.719**	**21.732**	**10.960**	**23.839**
- Weight	0.040	0.020	NA	2.400	**2.790**	0.190	NA	NA	0.080
-Weight∶year	**3.600**	**4.660**	NA	**8.240**	NA	**9.690**	**18.870**	**NA**	**20.530**
- FEC	**1.210**	**1.220**	NA	NA	NA	NA	0.060	NA	NA
-FEC∶year	**5.460**	**5.830**	NA	NA	NA	NA	**10.630**	**NA**	**11.120**
- Normalised *HL*	0.120	NA	0.140	NA	NA	NA	0.250	NA	0.160
-Normalised *HL*∶year	**4.450**	NA	**5.340**	NA	NA	NA	**10.130**	NA	**9.620**

MAM represents the variation explained by the minimum adequate model, composed of multiple covariates. The rows illustrate changes in the amount of variation explained when certain covariates are removed from the MAM. The colon between covariate terms indicates that their effects are being considered in an interaction with one another. Terms that explain a significant amount of variation are highlighted in bold. NA indicates where interactions were not present within the minimum adequate model.

**Table 4:** Percentage of variation explained by individual covariates in individual contributions to growth within female age classes.

	Lambs	Lambs	Lambs	Yearlings	Yearlings	Yearlings	Prime-aged	Prime-aged	Prime-aged	Senescent	Senescent	Senescent
Models	*p_t(i)_*	*S_t(i)_*	*F_t(i)_*	*p_t(i)_*	*S_t(i)_*	*F_t(i)_*	*p_t(i)_*	*S_t(i)_*	*F_t(i)_*	*p_t(i)_*	*S_t(i)_*	*F_t(i)_*
**MAM**	**16.43**	**16.12**	**20.72**	**16.4**	**16.51**	**16.08**	**13.5**	**22.02**	**4.142**	**22.69**	**23.47**	**14.32**
- Year	**5.987**	**5.717**	**15.119**	6.195	6.248	7.227	**6.889**	**16.021**	1.304	3.731	4.344	6.346
- Weight	0.230	0.230	**0.060**	**0.616**	**0.907**	0.062	0.010	NA	NA	0.250	**1.250**	0.106
-Weight∶year	**5.050**	**5.080**	**4.880**	9.066	7.273	**8.579**	**3.280**	**NA**	NA	7.660	6.530	7.907
- FEC	0.080	0.100	NA	NA	**1.400**	NA	**0.561**	**0.710**	0.069	**0.290**	**0.470**	NA
-FEC∶year	**4.150**	**4.230**	NA	NA	NA	NA	**3.130**	**2.320**	2.734	**7.830**	**7.740**	NA
- Normalised *HL*	NA	NA	NA	NA	NA	NA	NA	0.130	NA	NA	NA	NA
- Normalised *HL*∶year	NA	NA	NA	NA	NA	NA	NA	**3.290**	NA	NA	NA	NA

MAM represents the variation explained by the minimum adequate model, composed of multiple covariates. The rows illustrate changes in the amount of variation explained when certain covariates are removed from the MAM. The colon between covariate terms indicates that their effects are being considered in an interaction with one another. Terms that explain a significant amount of variation are highlighted in bold. NA indicates where interactions were not present within the minimum adequate model.

**Figure 2:**
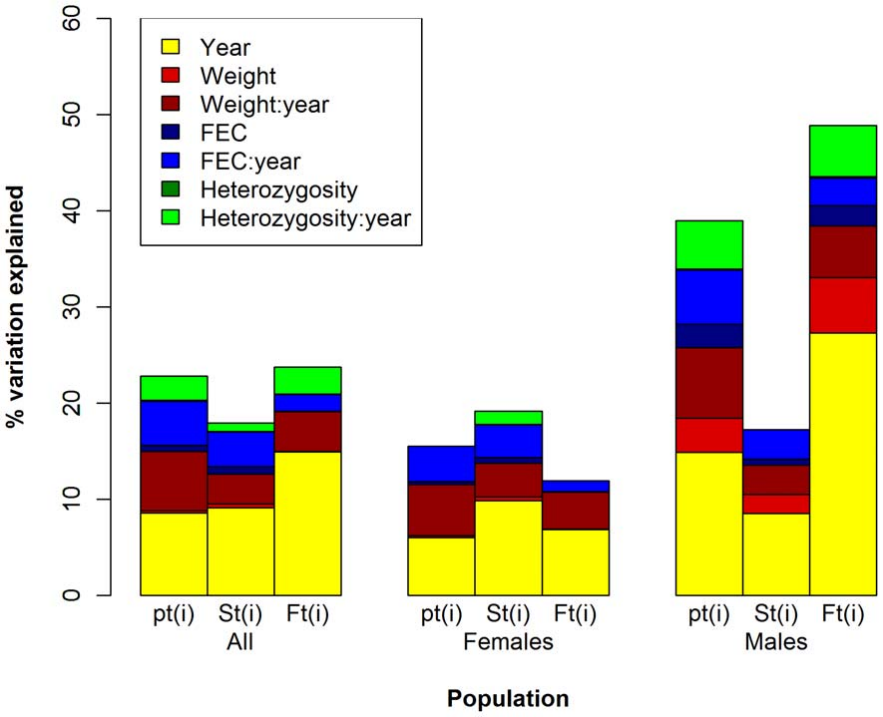
Variation in individual's contribution to population growth (*p_t(i)_*), via survival (*S_t(i)_*) and recruitment (*F_t(i)_*) explained by individual traits. The bars represent the total explained variation within *p_t(i)_*, *S_t(i)_* and *F_t(i)_* across different sections of the population (in the population as a whole, within females and within males). The different colours represent the proportion explained by individual covariates on their own or in an interaction with year. The colon between covariate terms indicates that their effects are being considered in an interaction with one another.

When we grouped individual contributions to population growth explained by individual traits within each age-class according to sex (Figure 2 and Table 5), normalised *HL* in an interaction with year explains approximately four times the amount of variation in both male *p_t(i)_* and fecundity compared to the variation explained in female survival. When it is considered on its own, it explains double the amount. Albeit more important in males, normalised *HL* still explains a minimal amount. Within male *p_t(i)_* and *F_t(i)_*, normalised *HL* explains approximately the same amount (approx. 0.1%), which is up to 30 and 50 times less variation respectively than when other traits are considered alone within the same groups. In an interaction with year, normalised *HL* explains approximately the same amount of variation in male *p_t(i)_* as FEC in an interaction with year (approx. 5%) and slightly less than weight in an interaction with year (being 7.33%). Within the contributions via male fecundity, normalised *HL* in an interaction with year accounts for approximately the same amount of variation as weight in an interaction with year (approx. 5%), and approximately 2% more variation than FEC in an interaction with year.

**Table 5:** Percentage of variation explained by individual covariates in individual contributions to growth within the population as a whole, and across females and males.

	All	All	All	Females	Females	Females	Males	Males	Males
Individual covariate terms	*p_t(i)_*	*S_t(i)_*	*F_t(i)_*	*p_t(i)_*	*S_t(i)_*	*F_t(i)_*	*p_t(i)_*	*S_t(i)_*	*F_t(i)_*
Year	8.565	9.106	14.95	6.034	9.858	6.866	14.897	8.528	27.309
Weight	0.275	0.425	0.038	0.197	0.392	0.043	3.557	1.975	5.734
Weight∶year	6.134	3.071	4.147	5.298	3.509	3.856	7.326	3.045	5.396
FEC	0.632	0.822	0.019	0.302	0.597	0.029	2.413	0.641	2.072
FEC∶year	4.616	3.581	1.733	3.72	3.392	1.137	5.641	3.063	2.901
Normalised *HL*	0.066	0.036	0.065	0	0.054	0	0.128	0	0.115
Normalised *HL*∶year	2.52	0.905	2.801	0	1.368	0	4.98	0	5.315
**Total variation explained**	**22.808**	**17.946**	**23.753**	**15.55**	**19.17**	**11.93**	**38.943**	**17.252**	**48.841**

MAM represents the variation explained by the minimum adequate model, composed of multiple covariates. The rows illustrate changes in the amount of variation explained when certain covariates are removed from the MAM. The colon between covariate terms indicates that their effects are being considered in an interaction with one another.

When the explanatory power of normalised *H* is grouped within females (Figure 2 and Table 5), it accounts for a minimal amount of variation in survival when analysed in an interaction with year (1.37%), and explains approximately three times less of the variation explained by both weight and FEC in an interaction with year. When normalised *HL* is considered on its own it explains an even more negligible amount of variation within female survival (0.05%), and accounts for the least amount of variation explained out of all traits. Although differences in individual weight and FEC on their own explain eight and twelve times more of the variation in female contributions via survival than normalised *HL*, their explanatory power is also very low (i.e. they explain less than 1%).

At the population level, normalised *HL* explains approximately the same low amount of variation in overall individual contributions to population growth (*p_t(i)_*) as that accounted for via survival and fecundity (approx. 0.05%; Figure 2 and Table 5). When considered in an interaction with year, normalised *HL* explains slightly more variation. Specifically, it accounts for about the same amount of variation in *p_t(i)_* and via fecundity (approx. 3%), whereas in survival it explains approximately three times less variation. Compared to the amount of variation explained by the other traits, normalised *HL* explains the least in *p_t(i)_* and via survival, both on its own and in an interaction with year. Within contributions via fecundity, normalised *HL* in an interaction with year explains 1% more variation than FEC in an interaction with year and when considered on its own it explains slightly more than both FEC and weight (up to 0.05% more).

## Discussion

In this article, we put forward two key findings. First of all, we demonstrate how the choice of method used to calculate multi-locus heterozygosity can influence ones results. We improve on previous methods by providing a normalising technique, which controls for variation in the number of loci genotyped between individuals. Secondly, we demonstrate that although heterozygosity influences some fitness components, most notably male reproductive success, in general it contributes very little to population growth in the Soay sheep of St. Kilda. We achieve this insight by extending a univariate approach, linking trait variation to individual contributions to population growth [13] into a multivariate framework.

Multi-locus heterozygosity quantities are frequently used to estimate how inbred or outbred an individual is, although recent research has queried how well they correlate with inbreeding coefficients [35], [36]. Nonetheless, multi-locus heterozygosity has been widely reported to influence fitness (e.g. [37]), even if the genetic processes it captures are not well understood. A range of multi-locus estimators have been developed, and although they are strongly correlated, the choice of estimator can influence results. We chose to work with *HL* as it determines the probability an individual is heterozygous given the alleles it carries and the frequency of those alleles within the population. Despite this, we still identified a problem with *HL*, and other measures of heterozygosity, as they all exhibit substantial heteroscedasticity as a function of the number of loci individuals are genotyped at, with much lower variation in heterozygosity among those individuals genotyped at a larger number of loci. Such heteroscedasticity could influence results, especially if the number of loci routinely genotyped increases with time within a study. We corrected for this heteroscedasticity by normalising the *HL* score within individuals genotyped at the same number of loci. This finding is crucial as it suggests that studies where individuals are genotyped at different numbers of loci across a population may be reporting biased mean heterozygosity values. If we did not normalise *HL* in this study, heterozygosity values from different individuals would not have been statistically comparable under the assumptions of normality. As a consequence, our understanding of the contribution of heterozygosity to population growth would have been flawed. To our knowledge, we are the first to consider this source of bias within heterozygosity calculations.

The second main finding of this study is that although heterozygosity has a weak role in population growth, there is considerable variation in the impact of heterozygosity on population dynamics within different gender and age groups. This is related to disparities in the importance of individual traits across population stages. Albeit weak, we found that individual traits explain approximately 2.5 times as much variation in contributions to population growth within males than within females. This difference is even more pronounced in the recruitment component (*F_t(i)_*) of contributions to population growth, where male individual differences account for approximately four times as much variation as in females. These results can be explained by the fact that individual traits are important in defining which males breed as they influence mating success during the rut [21]. In contrast, in prime-aged females, variation in size and FEC are of greater influence to their survival than reproduction. Since they do not need to compete for mates, females will invest more heavily in traits allowing them to survive the winter months, as well as over their pregnancy period [21]. The large contribution of the “year” term to variation in population growth estimates across all stages indicates that interannual differences explain a great deal of the variation in individual contributions to population growth. This highlights the importance of local environmental stochasticity within the dynamics of this population.

The components of the population where we find heterozygosity (defined by normalised *HL*) most strongly influences individual contributions to population growth are prime-aged females, male lambs, and adult males (composed of prime-aged and senescents). Of these, normalised *HL* accounts for approximately twice as much within male contributions to population growth as in females, specifically via adult reproductive success. Despite the relative importance of normalised *HL* in adult males, this effect does not leave a large signature on the population dynamics because it constitutes such a small fraction of the population. Heterozygosity in males determines which males successfully mate (in some years) even if this has no effect on the number of females that would become pregnant in the absence of heterozygous males. The importance of heterozygosity in reproductive success may differ between years on account of fluctuating selection, countervailing selection for different fitness components or frequency-dependent selection [19], [27]. Within females, normalised *HL* contributed little to population growth and typically much less than other measures of individual variation, such as body weight and FEC. As with all individual traits, when all females are considered together, normalised *HL* only contributes to survival. When each female age-class is considered separately, we find that this effect is experienced solely via prime-aged females.

Our findings add to the growing number of studies on heterozygosity-fitness correlations that show considerable variation in the strength of this relationship across species, populations and even between gender and age groups within a population. First of all, there has been evidence supporting class-specific effects of heterozygosity in populations of alpine marmots (*Marmota marmota*) and roe deer (*Capreolus capreolus*), with similarly low effect sizes [38], [39]. Previous studies of the Soay sheep population of Hirta have also found that heterozygosity explained little variation in parasite resistance [7] and neonatal birth weight and survival [32]. This finding is supported by a comprehensive meta-analysis of published and unpublished HFCs in animal populations (based on *MLH*, *Hs*, *IR*, d^2^ and standardised d^2^), which concluded that, generally, heterozygosity accounts for less than 1% of the variance in phenotypic characters associated with fitness [40]. In contrast, Sneddon *et al.* [8] found that in the subdesert mesite (*Monias benschi*) heterozygosity (measured by *H_s_* and IR) explained a considerable amount of variance in group territory size (r^2^ = approx. 60%), song structure (r^2^ = approx. 50% for males; 20% for females) and seasonal reproductive success (r^2^ = approx. 40%). Hanski and Saccheri (2006) also identified heterozygosity (*MLH*) as having an important role within population dynamics, accounting for 26% of the total deviance in a model developed for population extinction events within fragmented Glanville fritillary butterfly (*Melitaea cinxia*) populations. We propose that the range of discrepancies in the importance of heterozygosity for survival and breeding success across HFC analyses may reflect differences in recent immigration and mixing between populations as well as variation in selection pressures [40].

We extend a recently developed method [13] for linking individual and population level processes to gain insight into the role of heterozygosity in population dynamics. Using a statistical transformation of the homozygosity weighted by loci (*HL*) index, we show that the relative importance of heterozygosity in Soay sheep population growth differs markedly between sexes and age-classes. Overall, we find little evidence that heterozygosity influences population growth.

## Supporting Information

Table S1 Summary of linear models describing the association between normalised and non-normalised heterozygosity measures estimated using the selected panel of loci.
(DOCX)

## References

[1] Coltman DW, Bowen WD, Wright JM (1998). Birth weight and neonatal survival of harbour seal pups are positively correlated with genetic variation measured by microsatellites.. Proceedings of the Royal Society of London B.

[2] Coulson TN, Pemberton JM, Albon SD, Beaumont M, Marshall TC (1998). Microsatellites reveal heterosis in red deer.. Proceedings of the Royal Society of London B.

[3] Amos W, Wilmer J, Fullard K, Burg TM, Croxall JP (2001). The influence of parental relatedness on reproductive success.. Proceedings of the Royal Society of London B.

[4] Hansson B, Bensch S, Hasselquist D, Åkesson M (2001). Microsatellite diversity predicts recruitment of sibling great reed warblers.. Proceedings of the Royal Society of London B.

[5] Slate J, Kruunk LEB, Marshall TC, Pemberton J, Clutton-Brock TH (2000). Inbreeding depression influences lifetime breeding success in a wild population of red deer (Cervus elaphus).. Proceedings of the Royal Society of London B.

[6] Acevedo-Whitehouse K, Gulland F, Greig D, Amos W (2003). Disease susceptibility in California sea lions.. Nature.

[7] Coltman DW, Pilkington JG, Smith JA, Pemberton JM (1999). Parasite-mediated selection against inbred Soay sheep in a free-living, island population.. Evolution.

[8] Sneddon N, Amos W, Mulder RA, Tobias JA (2004). Male heterozygosity predicts territory size, song structure and reproductive success in a cooperatively breeding bird.. Proceedings of the Royal Society of London B.

[9] Koehn RK, Gaffney PM (1984). Genetic heterozygosity and growth rate in Mytilus edulis.. Marine Biology.

[10] Leary RF, Allendorf FW, Knudsen KL (1985). Inheritance of merisitc variation and the evolution of developmental stability in rainbow trout.. Evolution.

[11] Saccheri I, Kuussari M, Kankare M, Vikman P, Fortelius W (1998). Inbreeding and extinction in a butterfly metapopulation.. Nature.

[12] Hanski I, Saccheri I (2006). Molecular-level variation affects population growth in a butterfly population.. PloS Biology.

[13] Pelletier F, Clutton-Brock TH, Pemberton JM, Tuljapurkar S, Coulson TN (2007). The evolutionary demography of ecological change: linking trait variation and population growth.. Science.

[14] Hogg JT, Forbes SH, Steele BM, Luikart G (2006). Genetic rescue of an insular population of large mammals.. Proceedings of the Royal Society of London B.

[15] Vilà C, Sundqvist A-K, Flagstad Ø, Seddon J, Björnerfeldt S (2003). Rescue of a severely bottlenecked wolf (*Canis lupis*) population by a single immigrant.. Proceedings of the Royal Society of London B.

[16] Bensch S, Andrén H, Hansson B, Pedersen HC, Sand H (2006). Selection for heterozygosity gives hope to a wild population of inbred wolves.. PLoS ONE.

[17] Madsen T, Shine R, Olsson M, Wittzell H (1999). Restoration of an inbred adder population.. Nature.

[18] Aparicio JM, Ortego J, Cordero PJ (2006). What would we weigh to estimate heterozygosity, alleles or loci?. Molecular Ecology.

[19] Bancroft DR, Pemberton JM, Albon SD, Robertson A, MacColl ADC (1995). Molecular genetic variation and individual survival during population crashes of an unmanaged ungulate population.. Philosophical Transactions of the Royal Society of London B.

[20] Coulson TN, Benton TG, Lundberg P, Dall SRX, Kendall BE (2006). Estimating individual contributions to population growth: ecological fitness in ecological time.. Proceedings of the Royal Society of London B.

[21] Clutton-Brock TH, Pemberton JM, Clutton-Brock TH, Pemberton JM (2004). Individuals and populations.. Soay Sheep: Dynamics and Selection in an Island Population.

[22] Jewell PA, Milner C, Boyd JM (1974). Island survivors: the ecology of the Soay sheep of St. Kilda.

[23] Clutton-Brock TH, Price OF, Albon SD, Jewell PA (1992). Early development and population fluctuations in Soay sheep.. Journal of Animal Ecology.

[24] Clutton-Brock TH, Price OF, Albon SD, Jewell PA (1991). Persistent instability and population regulation in Soay sheep.. Journal of Animal Ecology.

[25] Grenfell BT, Price OF, Albon SD, Clutton-Brock TH (1992). Overcompensation and population cycles in an ungulate.. Nature.

[26] Clutton-Brock TH, Illius A, Wilson J, Grenfell BT, MacColl ADC (1997). Stability and instability in ungulate populations: an empirical analysis.. The American Naturalist.

[27] Pemberton JM, Smith JA, Coulson TN, Marshall TC, Slate J (1996). The maintenance of genetic polymorphism in small island populations: large mammals in the Hebrides.. Philosophical Transactions of the Royal Society of London B.

[28] Coulson TN, Catchpole EA, Albon SD, Morgan BJT, Pemberton JM (2001). Age, sex, density, winter weather, and population crashes in Soay sheep.. Science.

[29] Milner JM, Albon SD, Illius AW, Pemberton JM, Clutton-Brock TH (1999). Repeated selection of morphometric traits in the Soay sheep on St Kilda.. Journal of Animal Ecology.

[30] Catchpole EA, Morgan BJT, Coulson TN, Freeman SN, Albon SD (2000). Factors influencing Soay sheep survival.. Journal of Applied Statistics.

[31] Marshall TC, Slate J, Kruuk LEB, Pemberton JM (1998). Statistical confidence for likelihood-based paternity inference in natural populations.. Molecular Ecology.

[32] Overall ADJ, Bryne KA, Pilkington JG, Pemberton JM (2005). Heterozygosity, inbreeding and neonatal traits in Soay sheep on St Kilda.. Molecular Ecology.

[33] Ministry of Agriculture Fisheries and Food (1971). Manual of veterinary parasitological laboratory techniques.

[34] The R foundation for Statistical Computing (2009). R, version 2.9.0.. http://www.r-project.org/.

[35] Pemberton JM, Coltman DW, Bancroft DR, Smith JA, Paterson S, Clutton-Brock TH, Pemberton JM (2004). Molecular genetic variation and selection on genotype.. Soay Sheep: Dynamics and Selection in an Island Population.

[36] Queller DC, Goodnight KF (1989). Estimating relatedness using genetic markers.. Evolution.

[37] Coltman DW, Wilson K, Pilkington JG, Stear MJ, Pemberton JM (2001). A microsatellite polymorphism in the gamma interferon gene is associated with resistance to gastrointestinal nematodes in a naturally-parasitized population of Soay sheep.. The Journal of Parasitology.

[38] Cohas A, Bonnefant C, Kempenares B, Allainé D (2009). Age-specific effect of heterozygosity on survival in alpine marmots, *Marmota marmota*.. Molecular Ecology.

[39] Da Silva A, Gaillard J-M, Yoccoz NG, Hewison MAJ, Galan M (2009). Heterozygosity-fitness correlations revealed by neutral and candidate gene markers in Roe deer from a long-term study.. Evolution.

[40] Chapman JR, Nakagawa S, Coltman DW, Slate J, Sheldon BC (2009). A quantitative review of heterozygosity-fitness correlations in animal populations.. Molecular Ecology.

