# Supplementary material for: The Population Growth Consequences of Variation in Individual Heterozygosity
Source: PLoS One. 2011 May 18;6(5):e19667. doi: 10.1371/journal.pone.0019667 (PMC3097190; doi:10.1371/journal.pone.0019667)
Supplement: Table S1 — Summary of linear models describing the association between normalised and non-normalised heterozygosity measures estimated using the selected panel of loci. (DOCX) [file pone.0019667.s001.docx]

| **Heterozygosity measure A** | **Heterozygosity measure B** | **r²** | **F** | **p-value** |
| --- | --- | --- | --- | --- |
| MLH | Normalised *MLH* | 0.86 | 0.0002 | < 0.001 |
| *MLH* | *H_s_* | 0.93 | 0.0005 | < 0.001 |
| *MLH* | Normalised *H_s_* | 0.86 | 0.0003 | < 0.001 |
| *MLH* | HL | 0.98 | 0.00002 | < 0.001 |
| *MLH* | Normalised *HL* | 0.85 | 0.0002 | < 0.001 |
| Normalised *MLH* | *H_s_* | 0.86 | 0.0002 | < 0.001 |
| Normalised *MLH* | Normalised *H_s_* | 0.99 | 0.00006 | < 0.001 |
| Normalised *MLH* | HL | 0.86 | 0.0002 | < 0.001 |
| Normalised *MLH* | Normalised *HL* | 0.98 | 0.00002 | < 0.001 |
| *H_s_* | Normalised *H_s_* | 0.86 | 0.0003 | < 0.001 |
| *H_s_* | HL | 0.92 | 0.0005 | < 0.001 |
| *H_s_* | Normalised *HL* | 0.84 | 0.0002 | < 0.001 |
| Normalised *H_s_* | HL | 0.85 | 0.0002 | < 0.001 |
| Normalised *H_s_* | Normalised *HL* | 0.98 | 0.00001 | < 0.001 |
| HL | Normalised *HL* | 0.87 | 0.0003 | < 0.001 |
